# Constraint-based perturbation analysis with cluster Newton method: a case study of personalized parameter estimations with irinotecan whole-body physiologically based pharmacokinetic model

**DOI:** 10.1186/s12918-017-0513-2

**Published:** 2017-12-21

**Authors:** Shun Asami, Daisuke Kiga, Akihiko Konagaya

**Affiliations:** 1 Department of Computational Intelligence and Systems Science, Tokyo Institute of Technology, 4259 Nagatsuda-cho, Midori-ku, Yokohama-shi, Kanagawa 226-8503 Japan; 2 Department of Electrical Engineering and Bioscience, Waseda University, 2-2 Wakamatsu-cho, Shinjuku-ku, Tokyo, 162-8480 Japan; 3 School of Computing, Tokyo Institute of Technology, 4259 Nagatsuda-cho, Midori-ku, Yokohama-shi, Kanagawa 226-8503 Japan; 4 National Institute of Informatics, 2-1-2 Hitotsubashi, Chiyoda-ku, Tokyo, 101-8430 Japan

**Keywords:** Cluster Newton method, Constraint-based sensitivity analysis, PBPK models, Pharmacokinetics, Parameter estimation

## Abstract

**Background:**

Drug development considering individual varieties among patients becomes crucial to improve clinical development success rates and save healthcare costs. As a useful tool to predict individual phenomena and correlations among drug characteristics and individual varieties, recently, whole-body physiologically based pharmacokinetic (WB- PBPK) models are getting more attention. WB-PBPK models generally have a lot of drug-related parameters that need to be estimated, and the estimations are difficult because the observed data are limited. Furthermore, parameter estimation in WB-PBPK models may cause overfitting when applying to individual clinical data such as urine/feces drug excretion for each patient in which Cluster Newton Method (CNM) is applicable for parameter estimation. In order to solve this issue, we came up with the idea of constraint-based perturbation analysis of the CNM. The effectiveness of our approach is demonstrated in the case of irinotecan WB-PBPK model using common organ-specific tissue-plasma partition coefficients (Kp) among the patients as constraints in WB-PBPK parameter estimation.

**Results:**

We find strong correlations between age, renal clearance and liver functions in irinotecan WB-PBPK model with personalized physiological parameters by observing the distributions of optimized values of strong convergence drug-related parameters using constraint-based perturbation analysis on CNM. The constraint-based perturbation analysis consists of the following three steps: (1) Estimation of all drug-related parameters for each patient; the parameters include organ-specific Kp. (2) Fixing suitable values of Kp for each organ among all patients identically. (3) Re-estimation of all drug-related parameters other than Kp by using the fixed values of Kp as constraints of CNM.

**Conclusions:**

Constraint-based perturbation analysis could yield new findings when using CNM with appropriate constraints. This method is a new technique to find suitable values and important insights that are masked by CNM without constraints.

**Electronic supplementary material:**

The online version of this article (10.1186/s12918-017-0513-2) contains supplementary material, which is available to authorized users.

## Background

Despite considerable advances in biotechnology and in silico tools, effective drug development remains a challenging task. In fact, clinical development success rates are low and new drugs approved by the US Food and Drug Administration (FDA) have declined since the 1990s [1]. Especially, the success rates of oncology drugs are the lowest compared with non-oncology drugs; therefore, the costs of cancer drugs have been increasing dramatically over the last years. For example, the cost of ipilimumab, which has been approved in 2011 as a drug for melanoma in the United States of America is $120,000 per patient per year [2]. Considering the healthcare costs and the burden on patients, improvements of clinical development success rates and methods for a cost-efficient drug development are urgently needed.

Because efficacy or safety issues often make drug developments unsuccessful, individual optimized doses have the potential to help clinical development success [1]. Treatments with individually optimized doses indeed have improved the efficacy and safety compared to treatments with standard doses [3]. However, the determination of individually optimized doses involves very high costs because clinical trials are generally needed.

Most recently, the use of physiologically-based pharmacokinetic (PBPK) models is considered to be an efficient approach to personalized doses of drugs [4]. PBPK models have emerged as promising in silico tools for drug development. PBPK models are expected to predict pharmacokinetic properties that are useful for evaluating the effects of intrinsic (e.g., organ dysfunction, age, genetics) and extrinsic (e.g., drug- drug interactions, DDI) factors without the need for clinical studies [5]. Regulatory authorities such as the FDA recommend that pharmaceutical companies apply PBPK models to drug development [6, 7]. Actually, some drugs have been already approved solely based on PBPK models without accompanying clinical studies [8, 9], which indicates the usefulness of PBPK models for efficient drug development. Furthermore, PBPK models which are tailored to the individual patients will give us a lot of useful information for personalized medicine, such as individually optimized doses and treatments. Such PBPK models are considered effective tools to improve clinical development success rates and to decrease the costs of clinical development.

For the parameter estimation of PBPK models, Cluster Newton Methods (CNM) [10, 11] allow to find multiple solutions to an underdetermined inverse problem. The parameters of PBPK models are often estimated by conventional methods such as Gauss-Newton method or its derived algorithms. For a large number of parameters, however, these classical parameter estimation algorithms can be rather time-consuming, and are highly dependent on an initial parameter setting, which is often difficult due to the limited size of clinical data. Furthermore, the algorithms estimate only one set of optimized parameters while ignoring the diversity of the optimized parameter set resulting from the complexity. On the other hand, CNM can solve these problems by the following advantages reported in [11]. Firstly, CNM can accept a much broader range of the initial parameters of a PBPK model than the conventional algorithms. Thus, even if the suitable initial parameters are not known beforehand, CNM can find the proper parameters. Secondly, the computational costs are very low compared to other algorithms. Finally, the most important advantage is that CNM can provide a variety of optimized parameter sets which reproduce the observed clinical phenomena with a PBPK model. The parameter sets contain a lot of useful information that enable us to interpret the phenomena with higher confidence and extrapolate the obtained insights to new phenomena.

The previous reports applied CNM for estimating the parameters of PBPK models of irinotecan [10, 11]. As one of the key cancer drugs, irinotecan has been used for the treatment of many cancers worldwide. Irinotecan is also called CPT-11. It is metabolized to SN-38, NPC, and APC by carboxylesterase 2 (CES2) or CYP3A4, and moreover, NPC and SN-38 are metabolized to SN-38 and SN-38G by CES2 and UGT1A, respectively [12, 13]. Additionally, irinotecan pharmacokinetics are known to involve enterohepatic circulation (EHC). Thus, PBPK models of irinotecan used to be rather complicated, and the estimation of optimized parameters by the conventional methods was difficult.

Although the parameters of a simplified PBPK model of irinotecan were estimated by CNM [10, 11], the estimated parameters were obtained from general physiological data and from the average objective values of drug excretions in urine and feces of seven patients. Therefore, individual varieties such as sex, height and weight, and individual objective values of the eliminations could not be reflected, and correlations among the estimated parameters and individual varieties were not investigated.

In order to solve this issue, we here introduce a whole-body physiologically- based pharmacokinetic (WB-PBPK) model with personalized information and constrained-based perturbation analysis. A WB-PBPK model is an expanded PBPK model in which the organism is modeled as a circulatory system consisting of compartments that represent organs that are important for absorption, distribution, metabolism and elimination (ADME). Such models are quite useful for simulating personalized pharmacokinetics behavior because they can simulate personalized patient- specific variations (height, weight, age, sex) or physiological changes associated with disease-specific characteristics [14]. Thus, WB-PBPK models with many organs are increasingly important to develop personalized drugs and make personalized doses due to reflecting more individual varieties. WB-PBPK models generally require two types of input data: physiological parameters, such as blood flow and organ volumes, and drug-related parameters, such as clearance and tissue-plasma partition coefficients (Kp). The physiological parameters can be calculated based on personalized patient-specific data (height, weight, sex), and anatomic information [15]. The drug-related parameters are often estimated by the conventional methods. As mentioned in the previous section, however, the conventional methods have some problems for the parameter estimations of WB-PBPK models.

Although CNM can estimate the parameters in WB-PBPK models, it may cause overfitting when applying to individual clinical data such as urine/feces drug excretion for each patient without reflecting pharmacological or clinical knowledge. In general, Kp in the drug-related parameters depends on physical characteristics of drugs rather than individual differences between patients. Then, individual varieties of the drug-related parameters other than Kp may be masked when the parameters are estimated by the conventional CNM. In order to solve this issue, we came up with the idea of constraint-based perturbation analysis of the CNM. Our approach in more detail is explained by using a simple model in Additional file 1.

To be more precise, we estimate personalized drug-related parameters of irinotecan detailed WB-PBPK model with many organs and blood vessels by using CNM with personalized physiological parameters and individual objective values of eliminations. Additionally, the following steps for constrained perturbation analysis with CNM give us new insights. Firstly, we estimate individual values of all drug- related parameters including Kp for each organ by the conventional CNM without any constraints. Secondly, we estimate suitable values of Kp for each organ among all patients identically. Thirdly, we estimate personalized drug-related parameters, such as renal and liver clearances, by using CNM with constraints of the fixed values of Kp. Then, we observe the distributions of the personalized parameters with strong convergence in all patients and find considerable personalized factors when irinotecan is administered.

The novelty of our methodology is that we firstly show the way to deal with both common parameters and individual parameters among patients in WB-PBPK model. In our study, we found that a CNM with the constraints such as the fixed values of Kp can give us new insights which are masked in the conventional CNM without constraints. Thereby, we can better understand the correlations between pharmacokinetic parameters and individual varieties of physiological parameters and drug excretions.

## Methods

### Data source

We use reported individual data and pharmacokinetic information of irinotecan and its metabolites to estimate drug-related parameters and calculate personalized physiological parameters [16]. This report describes individual data such as sex, age, weight, body surface area, dose of irinotecan, and elimination ratio in urine and feces.

### WB-PBPK model structure of irinotecan

**Fig. 1:**
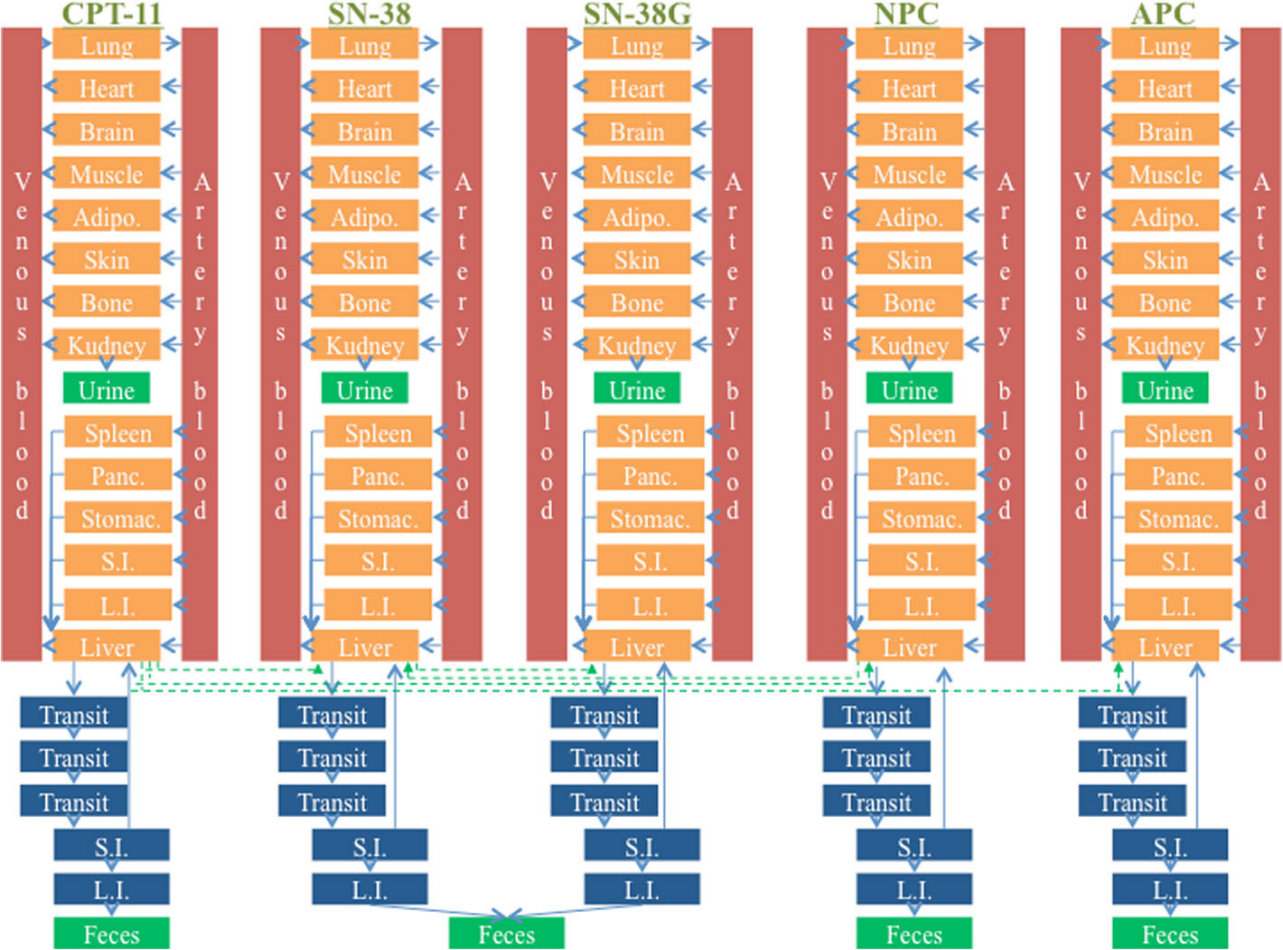
WB-PBPK model for irinotecan and the metabolites. We constructed a WB-PBPK model of irinotecan and the metabolites including blood circulatory compartments and elimination compartments. Blood circulatory compartments include venous and artery blood, lung, heart, brain, muscle, adipose (Adipo.), skin, bone, kidney, spleen, pancreas (Panc.), stomach (Stomac.), small intestine (S.I.), large intestine (L.I.) and liver. Elimination compartments include three biliary transit, S.I., L.I., urine and feces. Structure of the WB-PBPK model in each compound is described (Fig. 2)

**Fig. 2:**
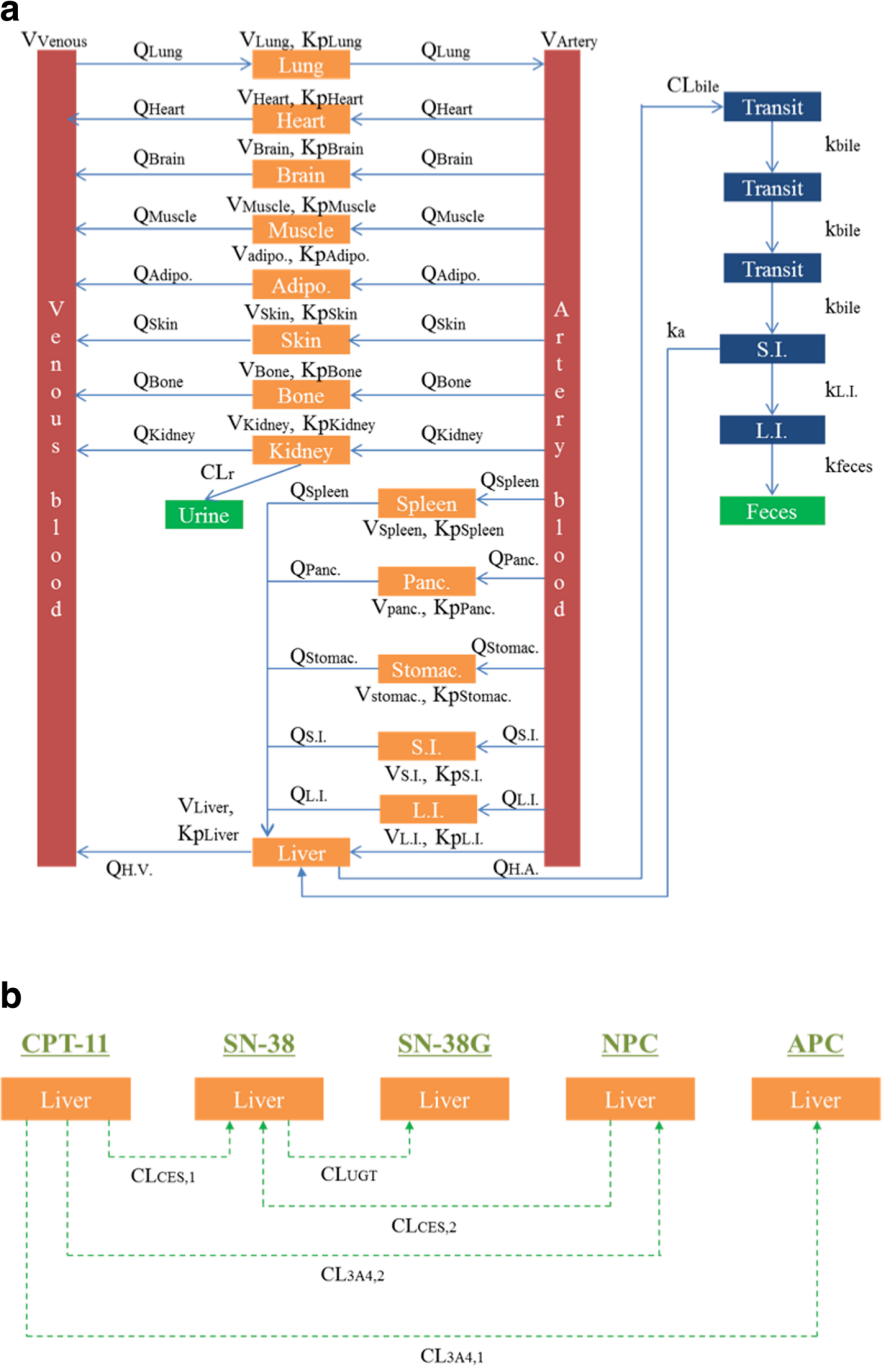
Parameters in the WB-PBPK model for irinotecan and the metabolites. Parameters are shown in the WB-PBPK model for irinotecan and the metabolites. **a** The kinds of parameters other than the metabolites in liver are same among irinotecan and the metabolites. Indicated are the blood flow (Q), the volume (V), the tissue-plasma partition coefficient (Kp), renal clearance (CL_r_), biliary clearance to transit compartment (CL_bile_), absorption rate constant (k_a_), kinetic constant for the transit in bile compartments to small intestine (k_bile_), kinetic constants for the transit from small intestine to large intestine (k_L.I._), kinetic constant for the transit from large intestine to feces (k_feces_), hepatic artery (H.A.) and hepatic vein (H.V.). The metabolic pathway of irinotecan and the metabolites in liver are represented **b**. Indicated are metabolic clearance of CPT-11 by CES2 to form SN-38 (CL_CES,1_), metabolic clearance of NPC by CES2 to form SN-38 (CL_CES,2_), metabolic clearance of CPT-11 by CYP3A4 to form APC (CL_3A4,1_), metabolic clearance of CPT-11 by CYP3A4 to form NPC (CL_3A4,2_) and metabolic clearance of SN-38 by UGT to form SN-38G (CL_UGT_)

The WB-PBPK model of irinotecan is constructed to estimate the drug-related parameters (Figs. 1 and 2. As mentioned in the introduction, irinotecan (CPT-11) is mainly metabolized into three compounds: SN-38, NPC and APC. Furthermore, SN- 38 and NPC are metabolized to SN-38G and SN-38, respectively. Therefore, these five compounds are included in our model. Our model is composed of 16 independent compartments for each compound, in which the following are included: venous and artery blood, lung, heart, brain, muscle, and adipose tissue; furthermore, skin, bone, kidney, spleen, pancreas, stomach, small intestine, large intestine and liver. Anatomically, the organs account for a large percentage in the human body [17]. Each compartment has an associated blood flow rate, volume and Kp (Fig. 2). Urine and feces are included as elimination compartments. SN-38G is assumed to be deconjugated to SN-38 by β-glucuronidase in intestinal microflora [18], and the sum of SN-38 and SN-38G are observable in feces. Additionally, three biliary transit compartments, small intestine, and large intestine are set to describe EHC. The dynamics of each compound are represented by differential equations in the supplementary material (Additional file 2). The differential equations are solved with MATLAB stiff ODE solver ODE15s [19].

### Parameter settings for WB-PBPK model of irinotecan

Patient characteristics, dose of irinotecan and physiological parameters are shown in Table 1. The patient characteristics and dose of irinotecan are based on the literature [16]. We excluded a bile duct cancer patient because the metabolic pathway of a patient with a bile duct t-tube as elimination pathway is very different from that of the other patients. The personalized physiological parameters are calculated by the reported method [15]. The method requires the following physiological information demographic information (sex, body weight and height) and organ information (mean of organ volumes and blood flows). As can be seen in Table 1, the reported patient information in [16] is used for the personalized information. The organ information is estimated by the reported anatomical information in [17].

**Table 1:** Patient characteristics, dose of irinotecan and physiological parameters

	Patient 1	Patient 2	Patient 3	Patient 4	Patient 5	Patient 6	Patient 7
Sex	Men	Men	Men	Men	Female	Female	Female
Age (years)	73	67	51	70	74	52	71
Height (cm)	171.6	169.4	182.3	182.5	170.6	169.4	188.6
Weight (kg)	109.1	89.1	78.6	98.2	88.0	52.7	83.2
BMI (kg/m2)^a^	37.1	31.0	23.7	29.5	30.2	18.4	23.4
Dose (μg/kg)	1000	1500	1700	1100	1400	2200	1400
Duration of infusion (min)	90	90	90	90	90	90	90
Volume (ml/kg)^b^							
Venous blood	12.8	15.6	18.7	14.9	12.3	20.4	14.0
Artery blood	8.1	9.8	11.7	9.4	7.7	12.8	8.8
Lung	11.6	14.1	16.9	13.5	11.9	19.7	13.5
Heart	3.8	4.5	5.4	4.4	3.9	6.4	4.4
Brain	13.8	16.9	19.2	15.4	15.4	25.7	16.3
Muscle	290.8	352.7	422.4	338.4	238.4	396.0	271.9
Adipose	469.0	360.6	240.0	391.2	516.4	210.8	452.6
Skin	41.7	46.0	50.3	40.3	38.1	49.1	40.7
Bone	106.3	128.9	154.4	123.7	107.3	178.2	122.3
Kidney	3.9	4.8	5.7	4.6	4.7	7.9	5.4
Spleen	2.2	2.7	3.2	2.5	2.6	4.3	2.9
Pancreas	1.7	2.1	2.5	2.0	2.0	3.3	2.3
Stomach	1.5	1.8	2.2	1.8	1.9	3.2	2.2
Small Intestine	6.5	7.9	9.5	7.6	8.2	13.6	9.3
Large Intestine	3.7	4.5	5.4	4.3	4.9	8.1	5.6
Liver	21.2	25.7	30.8	24.7	22.4	37.2	25.5
Blood flow Rate (ml/min/kg)^b^							
Lung	54.9	66.6	79.8	63.9	64.7	107.5	73.8
Heart	2.3	2.8	3.4	2.7	3.5	5.8	4.0
Brain	7.0	8.5	10.2	8.2	8.3	13.8	9.5
Muscle	9.9	12.1	14.4	11.6	7.8	13.0	8.9
Adipose	2.9	3.5	4.2	3.4	5.9	9.8	6.7
Skin	2.9	3.5	4.2	3.4	3.5	5.8	4.0
Bone	2.9	3.5	4.2	3.4	3.5	5.8	4.0
Kidney	11.9	14.5	17.3	13.9	13.2	21.9	15.0
Spleen	1.8	2.1	2.5	2.0	2.1	3.5	2.4
Pancreas	0.6	0.7	0.8	0.7	0.7	1.2	0.8
Stomach	0.6	0.7	0.8	0.7	0.7	1.2	0.8
Small Intestine	5.8	7.1	8.5	6.8	7.6	12.7	8.7
Large Intestine	2.3	2.8	3.4	2.7	3.5	5.8	4.0
Liver (Total)	14.9	18.1	21.7	17.3	18.7	31.1	21.4
Liver (Artery)	3.8	4.6	5.5	4.4	4.5	7.5	5.1

BMI, body-mass index

^a^BMI is calculated as weight(kg)÷height(m)2

^b^Volume and blood flow rate for each vessel and organ are calculated by using the work of Willmann et al. [15]

In Table 2, objective values for the parameter estimation are shown. The total elimination ratios of urine and feces are normalized to the ratios of the reported individual information [16]. The ratios of metabolism from CPT-11 to their metabolites and maximum serum concentrations (Cmax) of all compounds use the average values in [16]. Cmax of the metabolites are normalized to the amount of CPT-11.

**Table 2:** Objective values for each patient

ID	Parameters	Patient 1	Patient 2	Patient 3	Patient 4	Patient 5	Patient 6	Patient 7
Urinary elimination ratio (%)								
1	CPT-11	23.2	25.5	35.5	26.3	17.8	28.1	30.0
2	SN-38	0.4	0.5	0.7	0.5	0.3	0.5	0.6
3	SN-38G	3.1	3.4	4.8	3.6	2.4	3.8	4.0
4	NPC	0.1	0.2	0.2	0.2	0.1	0.2	0.2
5	APC	2.3	2.5	3.5	2.6	1.8	2.8	3.0
Fecal elimination ratio (%)								
6	CPT-11	45.3	43.5	35.4	42.7	49.6	41.4	39.9
7	SN-38 + SN-38G	11.9	11.4	9.3	11.2	13.1	10.9	10.5
8	NPC	1.9	1.8	1.5	1.8	2.1	1.7	1.7
9	APC	11.6	11.2	9.1	11.0	12.7	10.6	10.2
Cmax (μg/ml)								
10	CPT-11	1.53	1.53	1.53	1.53	1.53	1.53	1.53
11	SN-38	0.04	0.04	0.04	0.04	0.04	0.04	0.04
12	SN-38G	0.09	0.09	0.09	0.09	0.09	0.09	0.09
13	APC	0.19	0.19	0.19	0.19	0.19	0.19	0.19

Cmax, maximum serum concentrations

The total elimination ratio of urine and feces is normalized to the ratio of reported individual information. Ratios of metabolism from CPT-11 to its metabolites and Cmax of all compounds use average values in the report. Cmax of the metabolites are normalized to the amount of CPT-11

The initial ranges of drug-related parameters are shown in Table 3. The parameter ranges were initialized similarly to the approach reported in [11].

**Table 3:** Drug related parameters to estimate

ID	Parameters	Unit	Min	Max
1:5	Kp_Lung_	–	0.1	10
6:10	Kp_Heart_	–	0.1	10
11:15	Kp_Brain_	–	0.1	10
16:20	Kp_Muscle_	–	0.1	10
21:25	Kp_Adipose_	–	0.1	10
26:30	Kp_Skin_	–	0.1	10
31:35	Kp_Bone_	–	0.1	10
36:40	Kp_Kidney_	–	0.1	10
41:45	Kp_Spleen_	–	0.1	10
46:50	Kp_Pancreas_	–	0.1	10
51:55	Kp_Stomach_	–	0.1	10
56:60	Kp_Small intestine_	–	0.1	10
61:65	Kp_Large intestine_	–	0.1	10
66:70	Kp_Liver_	–	0.1	10
71	CL_r_(CPT-11)	ml/min/kg	0.1	10
72:75	CL_r_(metabolites)	ml/min/kg	0.01	1
76:80	CL_bile_	ml/min/kg	0.1	10
81	CL_CES,1_	ml/min/kg	0.1	10
82	CL_CES,2_	ml/min/kg	0.1	10
83	CL_3A4,1_	ml/min/kg	0.1	10
84	CL_3A4,2_	ml/min/kg	0.1	10
85	CL_UGT_	ml/min/kg	0.1	10
86:90	k_bile_	/min	0.001	0.1
91:95	k_a_	/min	0.0001	0.01
96:100	k_L.I._	/min	0.0001	0.01
101:105	k_feces_	/min	0.0001	0.01

Kp_Lung_, tissue-plasma partition coefficient of lungs; Kp_Heart_, tissue-plasma partition coefficient of heart; Kp_Brain_, tissue-plasma partition coefficient of brain; Kp_Muscle_, tissue- plasma partition coefficient of muscles; Kp_Adipose_, tissue-plasma partition coefficient of adipose; Kp_Skin_, tissue-plasma partition coefficient of skins; Kp_Bone_, tissue-plasma partition coefficient of bones; Kp_Kidney_, tissue-plasma partition coefficient of kidneys; Kp_Spleen_, tissue-plasma partition coefficient of spleen; Kp_Pancreas_, tissue-plasma partition coefficient of pancreas; Kp_Stomach_, tissue-plasma partition coefficient of stomach; Kp_Small intestine_, tissue-plasma partition coefficient of small intestine; Kp_Large intestine_, tissue-plasma partition coefficient of large intestine; Kp_Liver_, tissue-plasma partition coefficient of liver; CLr (CPT-11), renal clearance of CPT-11; CLr (metabolites), renal clearance of SN-38, SN-38G, NPC or APC, respectively; CL_bile_, biliary clearance to transit compartment; CL_CES,1_, metabolic clearance of CPT-11 by CES2 to form SN-38; CL_CES,2_, metabolic clearance of NPC by CES2 to form SN-38; CL_3A4,1_, metabolic clearance of CPT-11 by CYP3A4 to form APC; CL_3A4,2_, metabolic clearance of CPT-11 by CYP3A4 to form NPC; CL_UGT_, metabolic clearance of SN-38 by UGT to form SN-38G; k_bile_, kinetic constant for the transit in bile compartments to small intestine; k_a_, absorption rate constant; k_L.I._, kinetic constants for the transit from small intestine to large intestine; k_feces_, kinetic constant for the transit from large intestine to feces

### CNM method

In this study, Yoshida’s CNM algorithm with dS is used [11]. Firstly, a group of 1000 virtual samples as initial parameter sets is created by a random sampling from given parameter ranges (Table 3). The CNM algorithm has parameter dS to keep the diversity in population by means of creating a dividing point Xi with the ratio of dS: (1 − dS) where dS of 0 means the original CNM. The dS value is set 0.2 in our study, because the calculation converges adequately with this value. The first CNM is performed in 10 iterations to find a group of optimized parameter sets. After completing the parameter estimation, the calculated urinary and fecal ratios and Cmax of each compound are compared with the observed values. The comparisons of the observed values are based on the sum of squared residuals (SSR). Next, we select a group of some parameter sets based on small SSR value in all patients and calculate the median value of each tissue- plasma partition coefficient (Kp). The second CNM with the fixed values of Kp calculated is performed in 15 iterations to estimate the optimal parameters of the other drug-related parameters, such as renal and liver clearances. The number of virtual samples and value of dS are the same as those in the first CNM.

## Results and discussion

### Estimations of suitable values of Kp for irinotecan WB-PBPK regardless of individual varieties

**Fig. 3:**
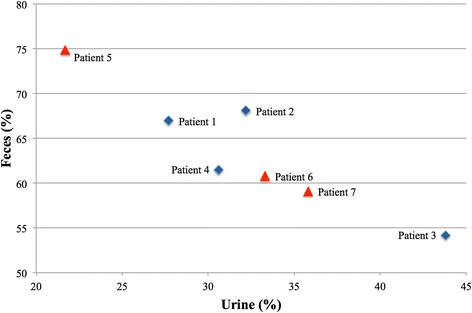
Individual varieties of elimination ratios from urine and feces. We visualized the individual varieties of elimination ratios from urine and feces. Blue diamonds and red triangles represent male and female patients, respectively

We estimated the individual varieties of some drug-related parameters by using WB-PBPK model with personalized physiological parameters and individual objective values of the eliminations. In our study, firstly, we estimated all drug-related parameters for each patient using conventional CNM, that is, CNM without constraints. Secondly, we estimated suitable Kp values among all patients identically by calculating the median of the estimated Kp values with low SSR (sum of squared residuals). Thirdly, we estimated the drug-related parameters other than Kp by using CNM with the fixed suitable values of each Kp, that is, using CNM with constraints. Finally, we observed correlations between the estimated drug-related parameters and individual varieties. We included more than 100 drug-related parameters and many compartments with 14 organs and 2 blood vessels for each substance in our WB-PBPK model (Figs. 1 and 2). Calculated personalized physiological parameters and reported individual doses of irinotecan are shown in Table 1. As reported in [16], there are varieties for the elimination ratios from urine or feces for the individual patients (Fig. 3).

The suitable values of Kp are estimated regardless of individual varieties by using CNM, since it is difficult for them to be estimated by classical parameter estimation algorithms due to the large number of Kp in our WB-PBPK model. As drug- related parameters, clearances in renal and liver, elimination rates to urine, feces, bile and large intestine, absorption rate of EHC, and Kp in each organ are involved. In general, Kp depends on physical characteristics of drugs rather than individual differences between patients. On the other hand, the clearances in renal and liver, the elimination rates and the absorption rate are significantly influenced by individual varieties such as age, weight, and body surface area. Parameter estimations in the WB- PBPK model were performed with a total of thirteen objective values: nine objective values of urinary and fecal accumulations and four objective values of Cmax of irinotecan and the metabolites (Table 2). Total elimination rate from urine and feces reflects individual observed data. Initial ranges of all drug-related parameters including Kp are shown in Table 3. The ranges are sufficiently large to include the real values for each parameter. Parameter estimations for all drug-related parameters were performed for each patient by using Yoshida’s CNM algorithm. The calculation time for the parameter estimations was relatively short, although in order to deal with quite complicated PBPK models, much more parameters must be estimated. The computation took less than 15 min with 10 iterations and 1000 virtual samples on a standard PC.

Next, we selected the estimated parameter sets with SSR (sum of squared residuals) 0.03 or less in all patients, because these parameter sets considerably reproduce the objective values. A total of 100 parameter sets can be selected in all patients. Then, median values of each Kp were calculated in the selected parameter sets (Additional file 3). Almost all values of Kp are around 1.0 and consistent with other PK model of irinotecan [20]; therefore, these values are probably appropriate.

### Estimations of parameters influenced individual varieties by using CNM with the fixed values of Kp

The other drug-related parameters that are influenced by individual varieties are estimated by using CNM with the median values of each Kp. Initial ranges of the parameters other than Kp are the same as the first CNM in Tables 3, and 1000 virtual samples were prepared. In the estimation, CNM required 15 iterations for parameter convergence. The parameter estimations were performed for each patient by using the same CNM algorithm as the first CNM. After the estimations, we calculated the coefficient of variation (CV) of the parameters and selected those parameters with CV = 0.3 or less, since these values showed strong convergences. As shown in Table 4, parameter #71 (Renal clearance of CPT-11), #76 (Liver clearance to bile of CPT-11), and #83 (Liver metabolism from CPT-11 to APC) show strong convergences in all patients. It appears that these parameters are important to reproduce the observed values. CVs of the parameters are slightly higher in patient 6 than in the other patients. Patient 6 has a lower body-mass index than the other patients, and suitable values of Kp may be different between this patient and the others. However, further investigation is needed to clarify the difference.

**Table 4:** Estimated parameters with CV 0.3 or less

ID: Parameters	Patient 1	Patient 2	Patient 3	Patient 4	Patient 5	Patient 6	Patient 7
71: CLr (CPT-11)	0.10	0.07	0.12	0.11	0.18	0.25	0.18
72: CLr (SN-38)	–	–	–	0.28	–	–	–
76: CLbile (CPT-11)	0.10	0.10	0.18	0.10	0.13	0.30	0.20
80: CLbile (APC)	–	–	0.26	0.27	–	–	–
82: CL_CES,2_	–	–	–	–	0.27	–	–
83: CL_3A4,1_	0.12	0.08	0.12	0.08	0.23	0.28	0.18

*CV* coefficient of variation, Bars represent CV is 0.3 over

### Distributions of strong convergent parameters

**Fig. 4:**
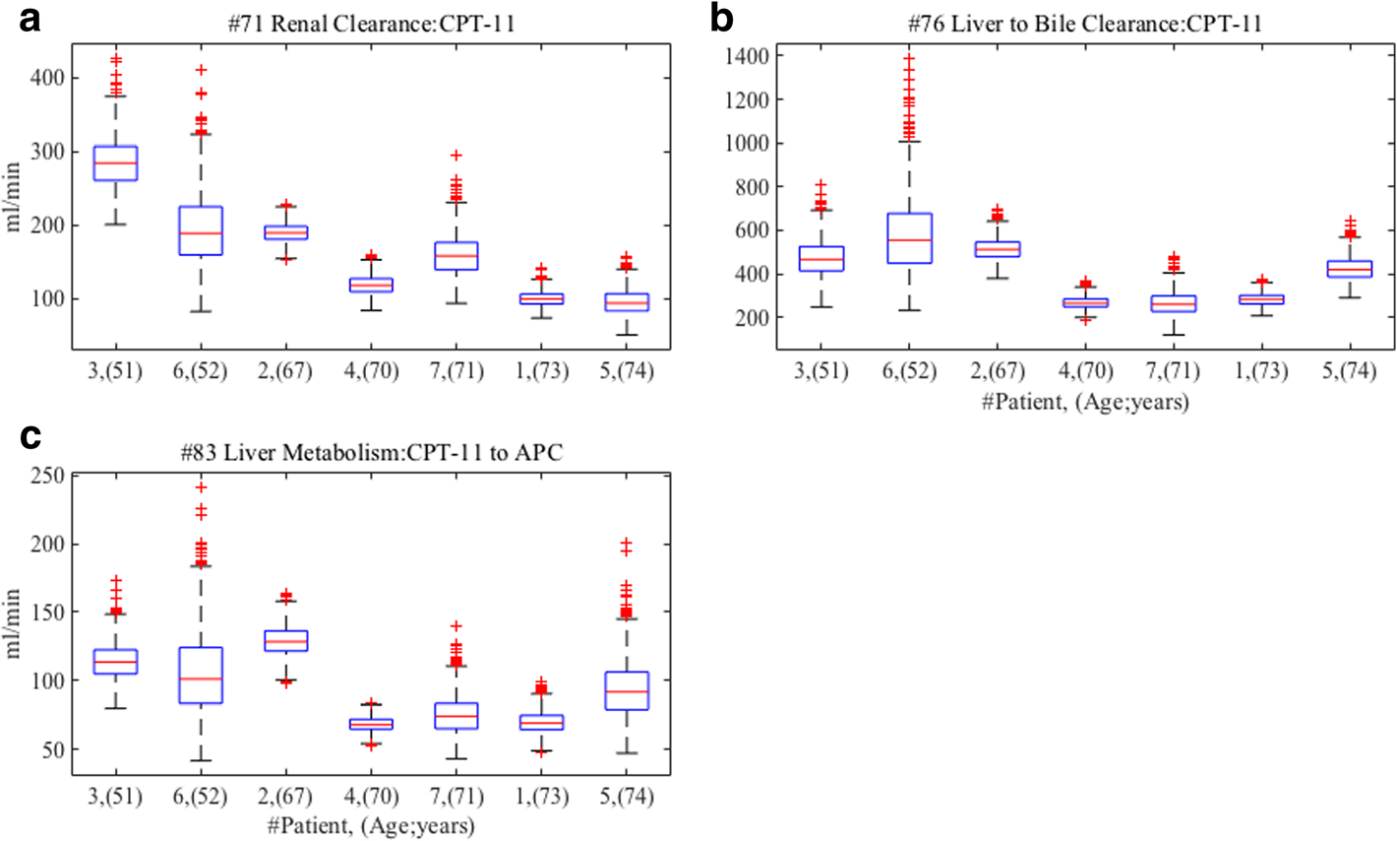
Distributions of parameters with strong convergences after second CNM with the fixed values of Kp. The distributions of the optimized values of the parameters with strong convergence are described after the parameter estimations by the CNM with the fixed values of Kp. The distributions of parameter #71 (renal clearance of CPT-11), #76 (liver clearance to bile of CPT-11), and #83 (liver metabolism from CPT-11 to APC) are shown in (**a**), (**b**) and (**c**), respectively

We observe distributions of the optimized values of the three parameters with strong convergence in all patients. As shown in Fig. 4, we find the distributions of parameter #71 (Renal clearance of CPT-11) are decreased in inverse proportion to age. On the other hand, #76 (Liver clearance to bile of CPT-11), and #83 (Liver metabolism from CPT-11 to APC) do not show the inverse proportion, however, elderly patients with 70 years and over decreased the distributions. In clinical practice, renal function is assessed by creatinine clearance calculated by Cockcroft-Gault equation including age as negative variable, and it has been reported that glomerular filtration rate decline with age [21]. Our results are partly in a good agreement with this clinical knowledge. According to the product information of irinotecan [22], the influence of renal impairment on pharmacokinetics of irinotecan has not been evaluated yet; therefore, the influence of changing renal clearance is not clear. Our finding possibly suggests that the administration of irinotecan needs to consider a patient’s age, since the renal clearance of CPT-11 may be decreased by age. Liver function indicated by parameter #76 (Liver clearance to bile of CPT-11) and #83 (Liver metabolism from CPT-11 to APC) do not show clear correlations with age, but the distributions of them are decreasing for elderly patients of 70 years and over. The results are consistent with the previous report that liver function such as clearance and metabolism by cytochrome is dramatically diminished in elderly patients of 70 years and over [23]. The administration of irinotecan should take into account a patient’s age, since the influence of elderly patients on pharmacokinetics of irinotecan has not also been clarified yet [22]. Our findings suggest that the dose of irinotecan should be adjusted for age. Although further investigations are needed to confirm the correlation between age, renal and liver function and pharmacokinetics of irinotecan, we can get new insights by observing the distributions of the parameters with strong convergences in optimized parameter sets obtained by CNM, especially when using with proper constraints.

### Differences in distributions of optimized parameters between by CNM with and without constraints

In our study, the first CNM was performed without constraints for all drug-related parameters to estimate Kp values for each patient. On the other hand, in the second CNM, we constrained the fixed Kp with the median values of the optimized parameter sets with low SSR (sum of squared residuals) in all patients, in consideration of pharmacokinetics and physiology.

**Fig. 5:**
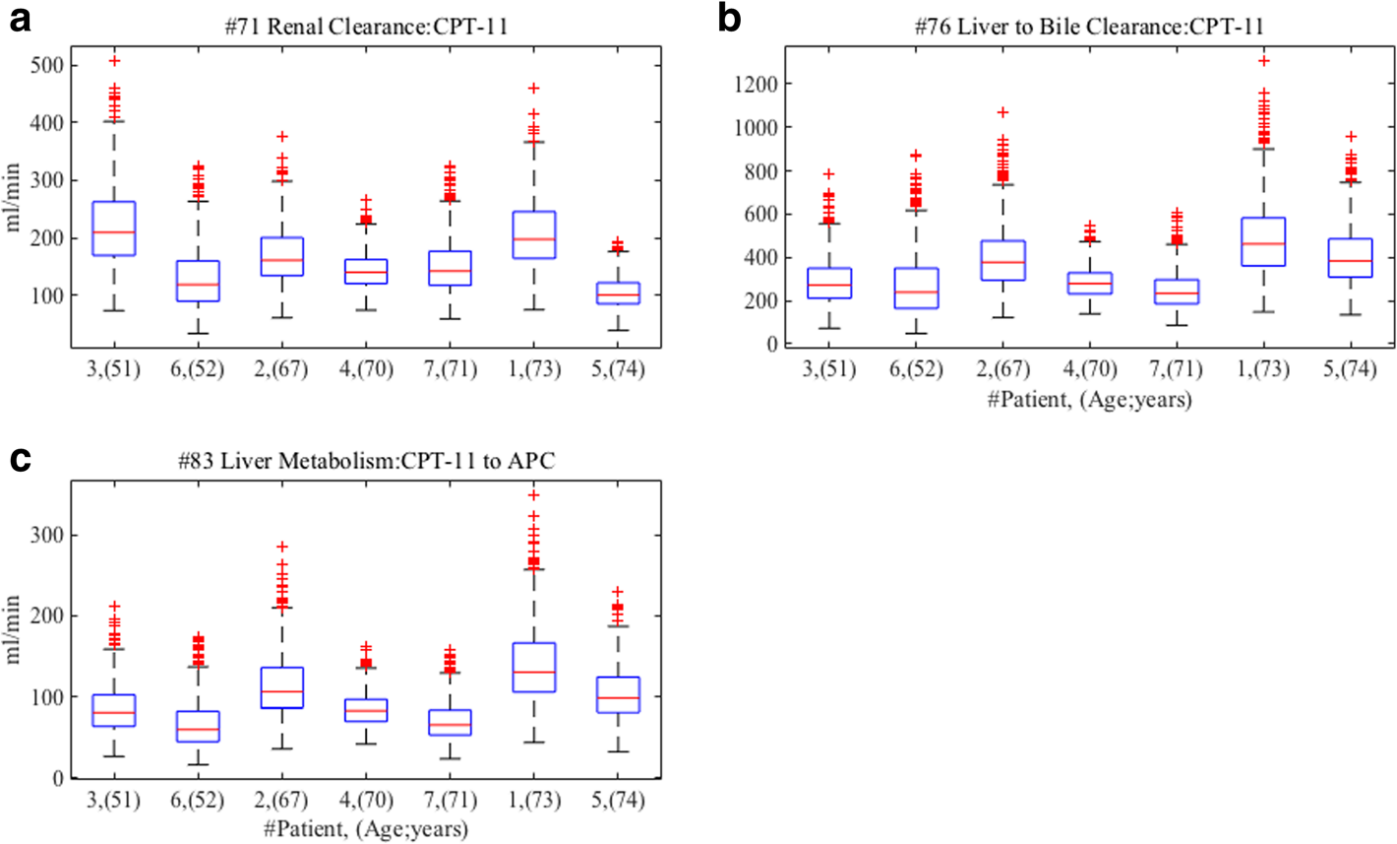
Distributions of parameters after the first CNM without constraints of Kp. The distributions of the optimized values of parameter #71 (renal clearance of CPT-11), #76 (liver clearance to bile of CPT-11), and #83 (liver metabolism from CPT-11 to APC) are described after the first CNM without constraints. The distributions of parameter #71, #76, and #83 are shown in (**a**), (**b**) and (**c**), respectively

**Fig. 6:**
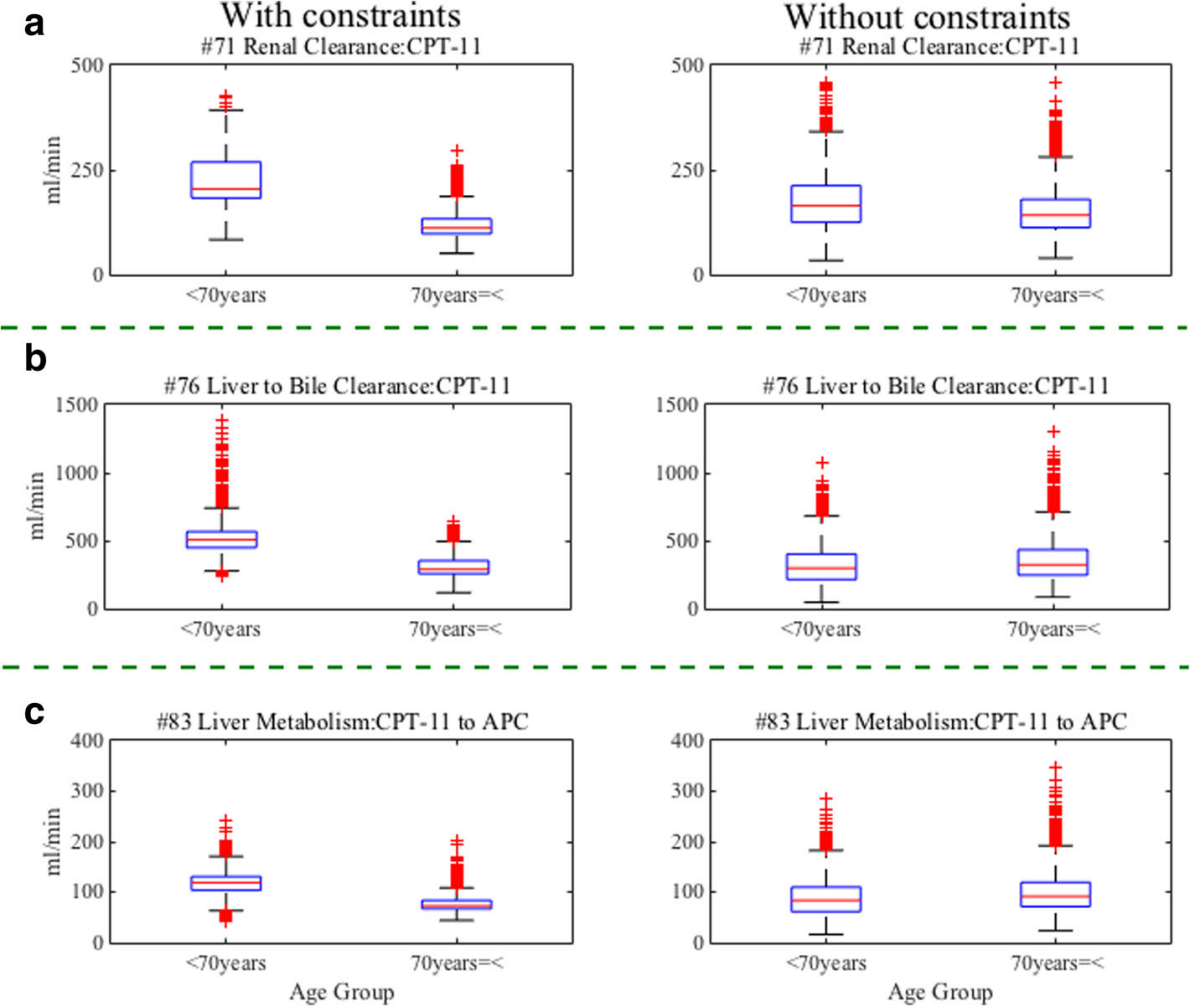
Comparing distributions of estimated parameters by age group between CNM with and without constraints. We observe the distributions of optimized values of the estimated parameter #71 (renal clearance of CPT-11), #76 (liver clearance to bile of CPT-11), and #83 (liver metabolism from CPT-11 to APC) in different age groups (below 70 years, and 70 years and over) between by the CNM with and without constraints. The distributions of parameter #71, #76, and #83 are shown in (**a**), (**b**) and (**c**), respectively. The distributions of the estimated parameters by the CNM without constraints are not different between both age groups. On the other hand, the distributions of the estimated parameters by the CNM with constraints are considerably lower in 70 years and over than in below 70 years

In summary, we observed differences in the distribution of optimized values of parameter #71, #76 and #83 between the first CNM without constraints and the second CNM with constraints. As shown in Fig. 5, the correlations between age and the parameters are not present in the first CNM without constraints. However, in the second CNM with constraints, the correlations are present as shown in Fig. 4. Furthermore, we divided all patients into two age groups (below 70 years and 70 years and over), and we observed distributions of optimized values of the parameters in the different age groups by both the CNM estimations with and without constraints (Fig. 6). The distributions of the estimated parameters by the CNM without constraints do not show any difference in both age groups. On the other hand, we can observe that the distributions of the parameters by the CNM with the constraints are considerably lower in the second group (70 years and over) than in the first group (below 70 years). It may appear that the Kp play a central role in adjusting the values of the other parameters so that the WB-PBPK model can reproduce the observed data for each patient, in other words, to mask true values of the parameters. This suggests that CNM with suitable constraints can give us new insights which are difficult to find when using observed data only. We strongly believe that CNM will be a more useful tool when using proper constraints based on knowledge of other disciplines as well as observed data.

## Conclusions

Constraint-based perturbation analysis with CNM is a powerful method to find masked relationships between parameters. In this study, we have successfully estimated personal parameter sets fitting to individual physiological parameters and excretions in irinotecan WB-PBPK model when assuming that Kp are the same among the patients. The results indicate strong correlations between age, renal clearance and liver function in the patients. Constraint-based perturbation analysis could present new findings when using CNM with suitable constraints, which should be guided by clinical background knowledge. Our methodology can be applicable to any PBPK models in which patient-dependent and patient-independent parameters are mixing together.

## Additional files

Additional file 1: A simple model for parameter estimations with and without constraint. We explain our approach of parameter estimation with or without constraint in this file. (PDF 180 kb)

Additional file 2: The ordinary differential equations in our WB-PBPK model nomenclature. This file shows the ordinary differential equations in our model. (PDF 86 kb)

Additional file 3: The median values of Kp from parameter sets with SSR 0.03 or less after first CNM. This file shows the median values of Kp from parameter sets with SSR 0.03 or less after first CNM. The values are fixed in second CNM with constraints. (XLS 46 kb)

## Abbreviations

Adipo: adipose
ADME: absorption, distribution, metabolism and elimination
BMI: Body-mass index
CES2: Carboxylesterase 2
CL_3A4,1_: Metabolic clearance of CPT-11 by CYP3A4 to form APC
CL_3A4,2_: Metabolic clearance of CPT-11 by CYP3A4 to form NPC
CL_bile_: Biliary clearance to transit compartment
CL_CES,1_: Metabolic clearance of CPT-11 by CES2 to form SN-38
CL_CES,2_: Metabolic clearance of NPC by CES2 to form SN-38
CLr (CPT-11): renal clearance of CPT-11
CLr (metabolites): renal clearance of SN-38, SN-38G, NPC or APC, respectively
CL_UGT_: metabolic clearance of SN-38 by UGT to form SN-38G
Cmax: Maximum serum concentrations
CNM: Cluster Newton Method
CV: Coefficient of variation
EHC: Enterohepatic circulation
FDA: Food and Drug Administration
H.A.: Hepatic artery
H.V.: Hepatic vein
k_a_: absorption rate constant
k_bile_: kinetic constant for the transit in bile compartments to small intestine
k_feces_: kinetic constant for the transit from large intestine to feces
k_L.I._: kinetic constants for the transit from small intestine to large intestine
Kp: tissue-plasma partition coefficient
Kp_Adipose_: tissue-plasma partition coefficient of adipose
Kp_Bone_: tissue- plasma partition coefficient of bones
Kp_Brain_: tissue-plasma partition coefficient of brain
Kp_Heart_: tissue-plasma partition coefficient of heart
Kp_Kidney_: tissue-plasma partition coefficient of kidneys
Kp_Large intestine_: tissue-plasma partition coefficient of large intestine
Kp_Liver_: tissue-plasma partition coefficient of liver
Kp_Lung_: tissue-plasma partition coefficient of lungs
Kp_Muscle_: tissue-plasma partition coefficient of muscles
Kp_Pancreas_: tissue-plasma partition coefficient of pancreas
Kp_Skin_: tissue-plasma partition coefficient of skins
Kp_Small intestine_: tissue-plasma partition coefficient of small intestine
Kp_Spleen_: tissue-plasma partition coefficient of spleen
Kp_Stomach_: tissue-plasma partition coefficient of stomach
L.I.: large intestine
Panc.: pancreas
PBPK: Physiologically based pharmacokinetic
Q_Adipose_: blood flow of adipose
Q_Bone_: blood flow of bones
Q_Brain_: blood flow of brain
Q_H.A._: blood flow of hepatic artery
Q_H.V_: blood flow of hepatic vein
Q_Heart_: blood flow of heart
Q_Kidney_: blood flow of kidneys
Q_L.I._: blood flow of large intestine
Q_Lung_: blood flow of lungs
Q_Muscle_: blood flow of muscles
Q_Panc._: blood flow of pancreas
Q_S.I._: blood flow of small intestine
Q_Skin_: blood flow of skins
Q_Spleen_: blood flow of spleen
Q_Stomac._: blood flow of stomach
S.I.: small intestine
SSR: sum of squared residuals
Stomac.: stomach
V_Adipose_: volume of adipose
V_Artery_: volume of artery
V_Bone_: volume of bones
V_Brain_: volume of brain
V_Heart_: volume of heart
V_Kidney_: volume of kidneys
V_L.I._: volume of large intestine
V_Liver_: volume of liver
V_Lung_: volume of lungs
V_Muscle_: volume of muscles
V_Panc._: volume of pancreas
V_S.I._: volume of small intestine
V_Skin_: volume of skins
V_Spleen_: volume of spleen
V_Stomac._: volume of stomach
V_Vein_: volume of vein
WB-PBPK: whole-body physiologically based pharmacokinetic
