# Supplementary material for: Constraint-based perturbation analysis with cluster Newton method: a case study of personalized parameter estimations with irinotecan whole-body physiologically based pharmacokinetic model
Source: BMC Syst Biol. 2017 Dec 21;11(Suppl 7):129. doi: 10.1186/s12918-017-0513-2 (PMC5763286; doi:10.1186/s12918-017-0513-2)
Supplement: Supplementary file 1 — A simple model for parameter estimations with and without constraint. We explain our approach of parameter estimation with or without constraint in this file. (PDF 180 kb) [file 12918_2017_513_MOESM1_ESM.pdf]

**Additional file 1: A simple model for parameter estimations with and without constraint**

[Setting]

We estimate parameters of “Y=AX+B” as a simple pharmacological model. Three patients are prepared. Clinically observed data of X and Y in each patient are shown in Table 1. Parameter A is not influenced by individual varieties. On the other hand, parameter B is influenced.

**Table S1 Individual clinical observed data of X and Y**

| | Patient 1 | Patient 2 | Patient 3 |
|---|---|---|---|
| X1, Y1 | 2, 100 | 3, 200 | 4, 4 |
| X2, Y2 | 7, 160 | 5, 300 | 8, 80 |
| X3, Y3 | 18, 350 | 15, 500 | 20, 200 |

[Parameter estimation with and without constraint]

When we estimate parameter A and B without constraint, the estimated parameters are in Table 2; they are shown as points on the solid lines in Figure 1. The values of parameters A and B are determined by each other. Then, individual characteristics of parameter B may be masked.

After the parameter estimation without constraint, we calculate the median of the values of parameter A among all patients. Next, we estimate parameter B by using the median value as common parameter A, that is, parameter estimation with a constraint. The estimated parameters are in Table 1 and are illustrated as points on the dotted lines in Figure 1. The estimated parameter B significantly reflects individual varieties, since X and Y are individually observed data, and parameter A is common.

**Table S2 Values of parameter A and B by parameter estimation with and without constraint**

| Parameter | Patient 1 | Patient 2 | Patient 3 |
|---|---|---|---|
| Without constraint | | | |
| A | 15.9 | 23.4 | 12.4 |
| B | 60.3 | 154.0 | -34.3 |
| With constraint of parameter A | | | |
| A (common) | 17.2 | 17.2 | 17.2 |
| B' | 48.3 | 201.2 | -85.8 |

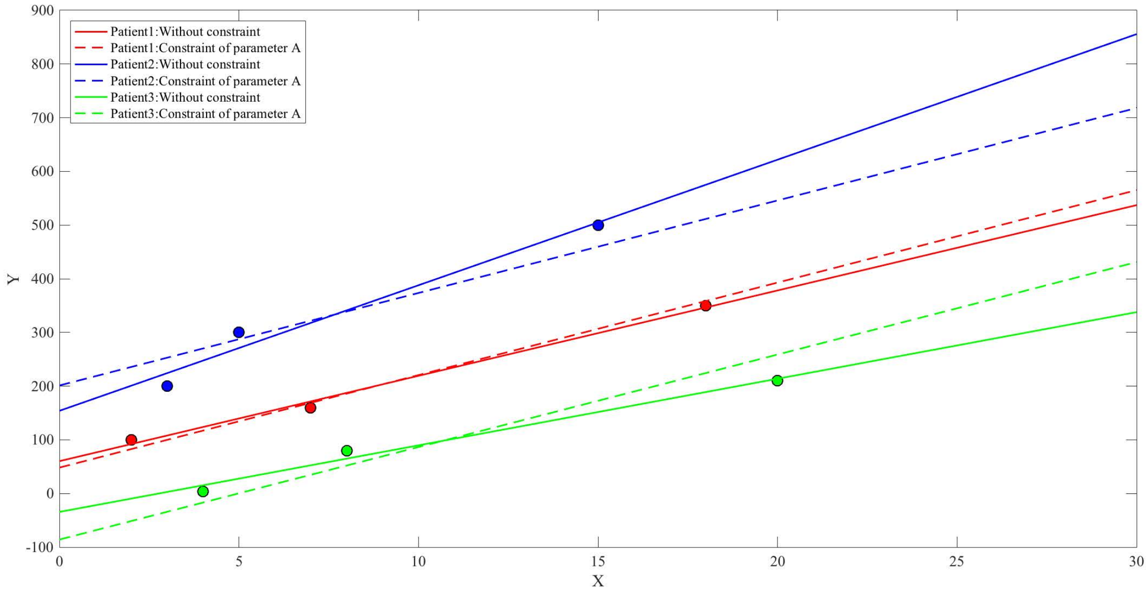


**Figure S1 Parameter estimation with and without constraint**
