# Supplementary material for: Constraint-based perturbation analysis with cluster Newton method: a case study of personalized parameter estimations with irinotecan whole-body physiologically based pharmacokinetic model
Source: BMC Syst Biol. 2017 Dec 21;11(Suppl 7):129. doi: 10.1186/s12918-017-0513-2 (PMC5763286; doi:10.1186/s12918-017-0513-2)
Supplement: Supplementary file 2 — The ordinary differential equations in our WB-PBPK model nomenclature. This file shows the ordinary differential equations in our model. (PDF 86 kb) [file 12918_2017_513_MOESM2_ESM.pdf]

Additional file 2: The Ordinary Differential Equations in our WB-PBPK model

Nomenclature

General. Q, blood flow rate; V, blood or organ weight; C, concentration; X, amount of compound; Kp, tissue-plasma concentration ratios; CL_r, renal clearance; CL_{bile}, biliary clearance to transit compartment; $CL_{CES,1}$, metabolic clearance of CPT-11 by CES2 to form SN-38; $CL_{CES,2}$, metabolic clearance of NPC by CES2 to form SN-38; $CL_{3A4,1}$, metabolic clearance of CPT-11 by CYP3A4 to form APC; $CL_{3A4,2}$, metabolic clearance of CPT-11 by CYP3A4 to form NPC; CL_{UGT}, metabolic clearance of SN-38 by UGT to form SN-38G; k_a, absorption rate constant; k_{bile}, kinetic constant for the transit in bile compartments to small intestine; $k_{L.I.}$, kinetic constants for the transit from small intestine to large intestine; k_{feces}, kinetic constant for the transit from large intestine to feces.

Subscripts. H.A., hepatic artery; H.V., hepatic vein; S.I., small intestine; L.I., large intestine. “i” corresponds to CPT-11 or one of the metabolites; 1, 2, 3, 4 and 5 represent CPT-11, SN-38, SN-38G, NPC and APC, respectively. “j” corresponds to Heart, Brain, Muscle, Adipose, Skin or Bone. “k” corresponds to Spleen, Pancreas, Stomach, Small

intestine or Large intestine.

Ordinary Differential Equations

The following ordinary differential equations for all patients are used in our study. Volume and blood flow rate in Table 1 are used for each patient.

(1) Compartments in blood circulatory system

- Venous blood

$V_{venous}(dC_{Venous,i}/dt) = Q_{Heart}C_{Heart,i}/Kp_{Heart,i} + Q_{Brain}C_{Brain,i}/Kp_{Brain,i} + Q_{Muscle}C_{Muscle,i}/Kp_{Muscle,i} + Q_{Adipose}C_{Adipose,i}/Kp_{Adipose,i} + Q_{Skin}C_{Skin,i}/Kp_{Skin,i} + Q_{Bone}C_{Bone,i}/Kp_{Bone,i} + Q_{Kidney}C_{Kidney,i}/Kp_{Kidney,i} + Q_{H.V.}C_{Liver,i}/Kp_{Liver,i} - Q_{Lung}C_{Venous,i}$

- Artery blood

$V_{Artery}(dC_{Artery,i}/dt) = Q_{Lung}C_{Lung,i}/Kp_{Lung,i} - (Q_{Heart} + Q_{Brain} + Q_{Muscle} + Q_{Adipose} + Q_{Skin} + Q_{Bone} + Q_{Kidney} + Q_{Spleen} + Q_{Pancreas} + Q_{Stomach} + Q_{S.I.} + Q_{L.I.} + Q_{H.A.})C_{Artery,i}$

- Lung

$V_{Lung}(dC_{Lung,i}/dt) = Q_{Lung}(C_{Venous,i} - C_{Lung,i}/Kp_{Lung,i})$

- Heart, Brain, Muscle, Adipose, Skin, Bone

$V_j(dC_{j,i}/dt) = Q_j(C_{Artery,i} - C_{j,i}/Kp_{j,i})$

- Kidney

$V_{Kidney}(dC_{Kidney,i}/dt) = Q_{Kidney}(C_{Artery,i} - C_{Kidney,i}/Kp_{Kidney,i}) - CLr_{,i}C_{Kidney,i}/Kp_{Kidney,i}$

- Spleen, Pancreas, Stomach, Small intestine, Large intestine

$V_k(dC_{k,i}/dt) = Q_k(C_{Artery,i} - C_{k,i}/Kp_{k,i})$

- Liver

$V_{Liver}(dC_{Liver,1}/dt) = Q_{H.A.}C_{Artery,1} + Q_{Spleen}C_{Spleen,1}/Kp_{Spleen,1} + Q_{Pancreas}C_{Pancreas,1}/Kp_{Pancreas,1} + Q_{Stomach}C_{Stomach,1}/Kp_{Stomach,1} + Q_{S.I.}C_{S.I.,1}/Kp_{S.I.,1} + Q_{L.I.}C_{L.I.,1}/Kp_{L.I.,1} + k_{a,1}X_{S.I.,1} - C_{Liver,1}(Q_{H.V.} + CL_{bile,1} + CL_{CES,1} + CL_{3A4,1} + CL_{3A4,2})/Kp_{Liver,1}$

$V_{Liver}(dC_{Liver,2}/dt) = Q_{H.A.}C_{Artery,2} + Q_{Spleen}C_{Spleen,2}/Kp_{Spleen,2} + Q_{Pancreas}C_{Pancreas,2}/Kp_{Pancreas,2} + Q_{Stomach}C_{Stomach,2}/Kp_{Stomach,2} + Q_{S.I.}C_{S.I.,2}/Kp_{S.I.,2} + Q_{L.I.}C_{L.I.,2}/Kp_{L.I.,2} + k_{a,2}X_{S.I.,2} + C_{Liver,1}CL_{CES,1}/Kp_{Liver,1} + C_{Liver,4}CL_{CES,2}/Kp_{Liver,4} - C_{Liver,2}(Q_{H.V.} + CL_{bile,2} + CL_{UGT})/ Kp_{Liver,2}$

$V_{Liver}(dC_{Liver,3}/dt) = Q_{H.A.}C_{Artery,3} + Q_{Spleen}C_{Spleen,3}/Kp_{Spleen,3} + Q_{Pancreas}C_{Pancreas,3}/Kp_{Pancreas,3} + Q_{Stomach}C_{Stomach,3}/Kp_{Stomac.,3} + Q_{S.I.}C_{S.I.,3}/Kp_{S.I.,3} +$

$Q_{L.I.}C_{L.I.,3}/Kp_{L.I.,3} + k_{a,3}X_{S.I.,3} + C_{Liver,2}CL_{UGT}/Kp_{Liver,2} - C_{Liver,3}(Q_{H.V.} + CL_{bile,3})/Kp_{Liver,3}$

$V_{Liver}(dC_{Liver,4}/dt) = Q_{H.A.}C_{Artery,4} + Q_{Spleen}C_{Spleen,4}/Kp_{Spleen,4} + Q_{Pancreas}C_{Pancreas,4}/Kp_{Pancreas,4} + Q_{Stomach}C_{Stomach,4}/Kp_{Stomach,4} + Q_{S.I.}C_{S.I.,4}/Kp_{S.I.,4} + Q_{L.I.}C_{L.I.,4}/Kp_{L.I.,4} + k_{a,4}X_{S.I.,4} + C_{Liver,1}CL_{3A4,2}/Kp_{Liver,1} - C_{Liver,4}(Q_{H.V.} + CL_{bile,4} + CL_{CES,2})/ Kp_{Liver,4}$

$V_{Liver}(dC_{Liver,5}/dt) = Q_{H.A.}C_{Artery,5} + Q_{Spleen}C_{Spleen,5}/Kp_{Spleen,5} + Q_{Pancreas}C_{Pancreas,5}/Kp_{Panc.,5} + Q_{Stomach}C_{Stomach,5}/Kp_{Stomach,5} + Q_{S.I.}C_{S.I.,5}/Kp_{S.I.,5} + Q_{L.I.}C_{L.I.,5}/Kp_{L.I.,5} + k_{a,5}X_{S.I.,5} + C_{Liver,1}CL_{3A4,1}/Kp_{Liver,1} - C_{Liver,5}(Q_{H.V.} + CL_{bile,5})/Kp_{Liver,5}$

(2) Elimination compartments

- Transit compartments

$dX_{Transit,1,i}/dt = C_{Liver,i}CL_{bile,i}/Kp_{Liver,i} - k_{bile,i}X_{Transit,1,i}$

$dX_{Transit,2,i}/dt = k_{bile,i}X_{Transit,1,i} - k_{bile,i}X_{Transit,2,i}$

$dX_{Transit,3,i}/dt = k_{bile,i}X_{Transit,2,i} - k_{bile,i}X_{Transit,3,i}$

- Small intestine

$$dX_{S.I.,i}/dt = k_{bile,i}X_{Transit,3,i} - (k_{a,i} + k_{L.I.,i})\, X_{S.I.,i}$$

- Large intestine

$$dX_{L.I.,i}/dt = k_{L.I.,i}X_{S.I.,i} - k_{feces,i}X_{L.I.,i}$$

- Urine

$$dX_{Urine,i} = CLr_{,i}C_{Kidney,i}/Kp_{Kidney,i}$$

- Feces

$$dX_{Feces,i} = k_{feces,i}X_{L.I.,i}$$
